# The Relationship of Fear of Death Between Neuroticism and Anxiety During the Covid-19 Pandemic

**DOI:** 10.3389/fpsyt.2021.648498

**Published:** 2021-04-20

**Authors:** Núria Pérez-Mengual, Inmaculada Aragonés-Barbera, Carmen Moret-Tatay, Adoración Reyes Moliner-Albero

**Affiliations:** ^1^Escuela de Doctorado, Universidad Católica de Valencia San Vicente Mártir, València, Spain; ^2^Faculty of Psychology, Universidad Católica de Valencia San Vicente Mártir, València, Spain; ^3^Dipartimento di Neuroscienze Salute Mentale e Organi di Senso, Department of Neuroscience, Mental Health and Sense Organs, Sapienza University of Rome, Rome, Italy

**Keywords:** fear to personal death, anxiety, personality, gender, COVID-19

## Abstract

After a lockdown, particularly one where human life is at risk, there are expected to be psychological consequences. The examination of personality traits, where different adaptative and non-adaptative behaviors in the face of adversity are expected, is our interest. The aim of this research was to analyze the role fear of personal death played during the Covid-19 outbreak in relation to personality and anxiety. The main results can be described as follows: women displayed higher scores on anxiety and fear of personal death; gender, fear of personal death, neuroticism, and extraversion predicted anxiety; in men, the fear of personal death mediated the relationship between neuroticism and anxiety.

## Introduction

The Coronavirus Disease 2019 (Covid-19) was first detected around December 2019 in the town of Wuhan, China. While a variety of restriction measures were employed by different countries, home isolation was one of the most common (1). Lockdown is understood as the restriction of movement with the aim to reduce the virus spread across a population. Side effects due to this drastic measure have been described in the literature (2). From a psychological level, the current situation generates concern and anxiety in individuals. Despite the fact that there is still not enough scientific literature to observe the long-term consequences, negative effects are anticipated (3). However, mortality information is widespread in the media and not surprisingly, feelings such as hopelessness, uncertainty, and fear of death are present in the population (4). These feelings can also be the consequence of perceived threat or triggering behaviors in search of self and community safety because life as we have known it up until now is changing. With time, they can become maladaptive, as well as result in hypervigilance and avoidance (5).

With regards to mental health, levels of anxiety, depression, and stress during the outbreak of Covid-19 have been described in the literature. According to Roma et al. (6), during a 2-month follow-up study carried out using an online questionnaire in Italy, an increase in stress and depression, but not in anxiety, was reported during confinement. Negative affect and detachment were also associated with higher levels of depression and stress. On the other hand, an online study by González-Sanguino et al. (7), described high levels of anxiety and depression, but also found a gender divide. They determined that women were more likely to report symptoms related to anxiety, depression, and post-traumatic stress disorders (8–10).

Regarding individual variables, it seems important to remember that personality traits have been associated with health behavior. Particularly, one of the most studied traits is neuroticism, which is often associated with inherent health concerns (11). Moreover, Mortensen et al. (12) showed that other features such as pleasantness, extraversion, and openness to experience are related to the prevention of infectious diseases and healthy behaviors. Furthermore, using the Big Five Traits model in a study aimed to address the protection factors for anxiety and depression during the Covid-19 pandemic in relation to personality traits, researchers found that extraversion, agreeableness, conscientiousness, and openness were negatively associated with generalized anxiety, but not neuroticism, which was positively correlated with generalized anxiety and depression. This is of interest as neuroticism is significantly related to generalized anxiety and depression (13).

In a prior study, Fitzpatrick et al. (14); Muris et al. (15) linked the neurotic personality to generalized anxiety. In this study, we will address this relationship when the fear of death manifests, as in the current pandemic, as well as explore what gender differences are expected. The literature has suggested that certain human behaviors could be explained through an attempt to receive psychological equanimity in the face of death (16). In this regard, outcomes inherent to individuals with a history of substance abuse employ suicide as a way to have control over their death, or fear of death (17). According to Ghazaei et al. (18), there is a paradoxical relationship between the evidence that death is inevitable and the instinctive desire to live. This work aims to measure the risk of these students based on religious orientation and fear of death. The results suggested that religious orientation and high-risk behavior was mediated by fear of death. Even if this mediation has not been examined in the current pandemic, to our knowledge, Liu et al. (19) highlight the importance of knowing the mediators between personality and stress to develop effective interventions designed to manage stress symptoms during a pandemic. Thus, the aim of this research is to study the mediation role of fear of death in the relationship between personality and generalized anxiety during the Covid-19 outbreak. It is hypothesized that fear of death mediates the prediction between neuroticism and anxiety, and differences between men and women are expected.

## Method

### Participants

A total of 303 Spanish participants volunteered to participate in the current study, from whom 40.9% were men and 59.1% were women. The age mean was 39.42 (SD = 12.01) with a range from 18 to 72. With regards to marital status, 31.7% were single, 62.7% were married or living with their partner, 1% widowed, and 4.6% were divorced. A total of 71.6% of the participants had to manage work with other familiar responsibilities, 10.6% were unemployed, 4% were retired, 8.6% were students, and 5.3% worked as a domestic helper without a fixed contract. The study was carried out in accordance with the Declaration of Helsinki and approved by the University ethical committee (UCV/2020-2021/041). Participants gave online consent to participate in the study.

### Materials

After a battery on sociodemographic data prepared for the present study, where the question “*I fear for my life because of Covid-19*,” was developed *in situ* with a similar procedure as Murphy and Moret-Tatay (8). This statement was answered in a Likert scale from 1 to 10 points. Next, the Generalized Anxiety Scale was employed [GAD-7; (20)] in its Spanish adaptation by Garcia-Campayo et al. (21). This is 7-item screening scale for anxiety disorders in the last 2 weeks, e.g., “*You have had difficulty relaxing*.” According McDonald's ω, the internal consistency was = 0.92.

Lastly, the BFI10 [Short Personality Scale; (22)] was also included in its Spanish adaptation (23). The questionnaire based on the MCF (Openness, Extraversion, Kindness, Responsibility, and Emotional Stability) presents an internal consistency between 0.80 and 0.90 in the literature. The BFI10 has 10 short statement items, two for each dimension, e.g., *I see myself as an extraverted person*. According McDonald's ω, the internal consistency was = 0.42. Even if this was not an optimal value, researchers have reported similar results in the literature. As stated by Gosling et al. (24) that criteria like alpha and clean factor structures are only meaningful to the extent they reflect improved validity and a more appropriate index would be test-retest reliability.

### Procedure

The study was carried out during the last week of December 2020. A cross-sectional design, relying on online self-reports under a snowball sampling was employed. Thus, sampling procedure was incidental, under a small pool of initial informants who shared the online questionnaire in their institutional and social networks. The questionnaire was self-administered, and participants were volunteers who completed the necessary informed consent documentation.

### Data Analysis

Data analysis was performed using JASP (Version 0.12.2) [Computer software]. Descriptive analysis and correlational analysis under Pearson coefficient were carried out. Moreover, a *t*-test for independent samples across gender, as well as its effect size under Cohen's d', and a linear regression model on the prediction of anxiety were carried out. This procedure was also taken into consideration as different confounders might occur, but it also to previously examine and avoid collinearity across variables. This involved procedures such as evaluating outliers or the independence of linearly uncorrelated residuals as depicted in previous literature (25). The statistical analysis for the mediation model was performed using SPSS 22 (IBM) under the Process macro for SPSS (26) to test the hypothesis that death fear mediates the effect of personality on anxiety. This is described as the Model 4 in the Hayes' Process Marco. In this way, Regression-based procedures were executed employing bootstrapping procedures using 10,000 samples (27, 28). The average estimate for indirect effect from the bootstrap samples, standard error, and lower and upper confidence limits were calculated. In this way, if the 95% confidence limits include zero, the indirect effect test is not significant (29).

## Results

Descriptive analysis was carried out on the variable of interest for the whole dataset, as well as Pearson's correlations, as depicted in Table 1. The strongest relationships were found between neuroticism and anxiety, and fear of death and age. A student's *t*-test between gender was carried out for the variables under study. As expected, the scores were higher for women than men and these statistical differences were found for fear of death (M_ean_ = 4.58 vs. M_ean_ =6.02; *t*_(301)_ = 5.11; *p* < 0.001; Cohen's *d* = −0.59) Conscientiousness (M_ean_ = 7 vs. M_ean_ = 7.88; *t*_(301)_ = 4.49; *p* < 0.001; Cohen's *d* = −0.46) and Anxiety (M_ean_ = 7.69 vs. M_ean_ = 10.21; *p* < 0.001; *t*_(301)_ = 3.99; Cohen's *d* = –0.52).

**Table 1:** Descriptive statistics on the variables under study and correlations among each other's.

	**Mean (SD)**	**1**	**2**	**3**	**4**	**5**	**6**	**7**	**8**
Age (1)	39.42 *(12.01)*	–							
Death fear (2)	5.43 *(2.52)*	0.19^**^	–						
Anxiety (3)	9.18 *(5.52)*	−0.09	0.41^**^	–					
Openness (4)	7.50 *(1.52)*	0.13^*^	−0.05	−0.15^**^	–				
Extraversion (5)	7.24 *(1.98)*	0.03	0.02	−0.17^**^	0.31^**^	–			
Conscientiousness (6)	7.52 *(1.74)*	0.21^**^	0.04	−0.17^**^	0.12^*^	0.17^**^	–		
Agreeableness (7)	8.08 *(1.52)*	0.09	−0.09	−0.25^**^	0.19^**^	0.06	0.30^**^	–	
Neuroticism (8)	4.94 *(2.01)*	−0.19^**^	0.21^**^	0.60^**^	−0.17^**^	−0.17^**^	−0.40^**^	−0.50^**^	–

* *p < 0.05*,

** *p < 0.01*.

A linear regression was carried out on the prediction of anxiety and on the target variables under the study predictors. The gender variable was included in the analysis as a dummy variable. The Adjusted *R*^2^ for the whole data set was 0.47, and the resulting model was statistically significant; *F*_(8_, _302)_ = 32.72; MSE = 543.15; *p* < 0.001. **Table 3** depicts the coefficients and variables included in the model. Table 2 depicts the regression coefficients for the model. These indicated that the highest coefficient to predict anxiety was neuroticism, followed by fear of death, gender, and extraversion.

**Table 2:** Regression coefficients on the prediction of anxiety.

**Model**	**B**	**SE**	**β**	***t***	***p***
(Intercept)	−1.380	2.600		−0.531	0.596
Death fear	0.615	0.102	0.280	6.020	<0.001
Extraversion	−0.257	0.127	−0.092	−2.015	0.045
Conscientiousness	0.072	0.157	0.023	0.459	0.647
Agreeableness	0.122	0.183	0.034	0.665	0.507
Neuroticism	1.449	0.152	0.527	9.528	<0.001
Openness	−0.075	0.166	−0.021	−0.453	0.651
Age	−0.028	0.021	−0.062	−1.364	0.174
Gender	1.301	0.523	0.116	2.489	0.013

**Figure 1:**
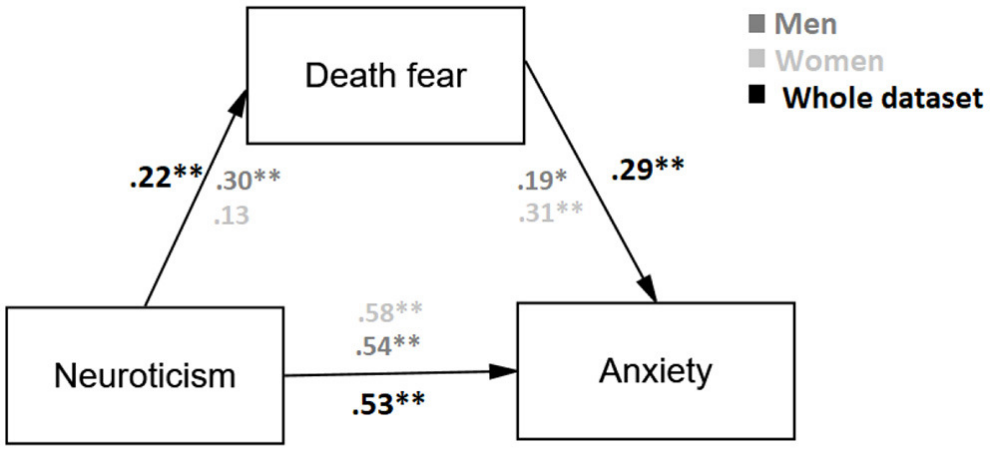
Estimated model, where fear of death mediate the relationship between personality and anxiety (**p < 0.01;*p < 0.5).

Lastly, a mediation analysis was carried out. Personality was considered an independent variable; fear of death was considered a mediator, and anxiety was a dependent variable. As expected, the only trait of personality that had statistical significance was Neuroticism. This model was conducted between women and men of the whole data set (see Figure 1). Table 3 depicts the confidence interval (CI) at 95% was statistically significant and a confidence interval that does not include the zero value.

**Table 3:** Effect of X (Neuroticism) on Y (Anxiety), Standard error (SE), statistical significance, and lower and upper (LLCI and ULCI) levels.

**Model**	**X on Y effect**	**Effect**	**SE**	***t***	***p***	**LLCI**	**ULCI**
Whole dataset	Total	0.60	0.04	13.90	<0.001	0.51	0.68
	Direct	0.54	0.07	12.07	<0.001	0.44	0.62
	Indirect	0.06	0.02	–	–	0.02	0.11
Men	Total	0.60	0.06	9.47	<0.001	0.47	0.72
	Direct	0.54	0.07	7.27	<0.001	0.39	0.69
	Indirect	0.05	0.02	–	–	0.01	0.13
Women	Total	0.58	0.06	9.84	<0.001	0.46	0.69
	Direct	0.54	0.05	9.41	<0.001	0.43	0.65
	Indirect	0.04	0.02	–	–	−0.001	0.09

## Conclusions and Discussion

After lockdown, psychological consequences in the population are expected. According to literature of the most common mental symptoms in the general population, are related to symptoms of depression. The traits that are of interest for the current the pandemic include being a woman, young age, being a student, a low level of education or even “overeducation” (30, 31). Overeducation is defined as having a level of education higher than what is required for a specific job (32). Since personality traits are of interest, it is important to note that we expect different adaptative and non-adaptative behaviors to face adversity (12, 15). The aim of this research was to analyze the role of fear of personal death during the Covid-19 outbreak in the relationship between personality and anxiety. Findings can be described as followed; women displayed higher scores on anxiety and fear of personal death; gender, fear of personal death, neuroticism, and extraversion all predicted anxiety; in men, but not women, the fear of personal death mediated the relationship between neuroticism and anxiety.

Current results support that neuroticism, as a personality trait, is significantly associated with anxiety during the Covid-19 outbreak and other factors such as worry related to fear (14, 33). Moreover, a mediation effect was found for men, suggesting that this group might experience higher anxiety when comparing fear of death found in women. Eshbaugh and Henninger (34) pointed out that women report a greater level of anxiety related to death than men do, however, as also indicated by the authors, mediation analysis are lacking in the field. Thus, the case of women would be more complex. This might depict a vulnerable situation for this profile; that is why these results might be of interest for both theoretical and applied levels of personality traits and death awareness.

According to Ahorsu et al. (35), infectious disease such as Covid-19 lead to psychosocial challenges including stigmatization and/or discrimination. Particularly, men with the high levels of fear, may not think clearly and rationally when reacting to Covid-19 because of high levels of anxiety. Thus, we consider that understanding its role might shed light for health programs from a holistic perspective. Furthermore, it has also been hypothesized that women often have the role of providing care for older relatives and in the raising and care of children, attributed in most cases to the gender role, which leads to an increase in workload. Thus, the “pandemic fear” might involve more agents in this group, as depicted in previous literature (36). Blurring situation between work, health, and life obligations might buffer fear to personal death in this profile, but its relationship might be even more complex for women. In fact, previous literature found that anxiety sensitivity and panic-related appraisals mediated gender differences in phobic avoidance (37). Moreover, González-Sanguino et al. (7), in a study assessing the emotional consequences of the COVID-19 pandemic, concluded that women were more likely to develop anxiety, depressive and post-traumatic stress symptoms. Pappa et al. (9) also found similar results regarding gender, observing that women who were dedicated to the field of health, obtained higher results than men who worked in the same occupational field. These gender differences can be considered as a way of gender inequality.

On the other hand, and according to the traditional Terror Management Theory (TMT), which is not as cited or popular of a theory today, self-preservation gives rise to a certain existential terror that can act raising anxiety to attitudes and cultural aspects or shared beliefs about reality, as well as other variables (38, 39). The emergence of different and, most of the time, polarized views on the Covid-19 could describe the dynamic interaction between individual and cultural differences which might also be related, among others, to gender roles (40). In this scenario, the main implications of the current results might be linked to understanding individual differences. These can be crucial and helpful recognizing the Covid-19 health footprint.

As differences between gender on the role of fear of death have been found, future lines of research should address differences between emotional and problem focus coping when facing adversity, since gender differences have been described in previous literature (31). This might depict differences strategies that might interfere with gender that are of interest in order to avoid potential confounders in a mediation analysis. The main limitation that arises in this study is that the sample was selected through non-probability sampling under a cross-sectional design, which can introduce distortions in the results. Moreover, data was recruited in a self-informed way. Furthermore, validated tools in assessing fear of death were not employed. In this way, future lines of research should adapt tools such as the “Fear of COVID-19 Scale” (35) in the Spanish population. One should not ignore fear of Covid-19 when studying fear of death. Thus, we expect that these results will be a starting point for future research along these lines. Another variable of interest would be if participants have experienced the loss of relatives/friends because of Covid-19, as it might increase the psychological suffering and the process of fear of personal death (41). Lastly, specific mental health conditions were not recruited in the current study since a higher susceptibility to stress compared with the general population might occur, this is also of interest for further research in the field (42).

## Data Availability Statement

The raw data supporting the conclusions of this article will be made available by the authors, without undue reservation.

## Ethics Statement

The studies involving human participants were reviewed and approved by UCV. The patients/participants provided their written informed consent to participate in this study.

## Author Contributions

All authors listed have made a substantial equally, direct and intellectual contribution to the work, and approved it for publication.

## Conflict of Interest

The authors declare that the research was conducted in the absence of any commercial or financial relationships that could be construed as a potential conflict of interest. The reviewer PR declared a shared affiliation with one of the authors CM-T, to the handling editor at time of review.
